# Cerebral Arterial Pulsatility and Global White Matter Microstructure Impact Spatial Working Memory in Older Adults With and Without Cardiovascular Risk Factors

**DOI:** 10.3389/fnagi.2020.00245

**Published:** 2020-08-07

**Authors:** Alexander C. Conley, Frini Karayanidis, Todd A. D. Jolly, Meng-Heng Yang, Shulan Hsieh

**Affiliations:** ^1^Department of Psychiatry, Center for Cognitive Medicine, Vanderbilt University Medical Center, Nashville, TN, United States; ^2^Functional Neuroimaging Laboratory, School of Psychology, Faculty of Science, University of Newcastle, Newcastle, NSW, Australia; ^3^Priority Research Centre for Stroke and Brain Injury, University of Newcastle, Newcastle, NSW, Australia; ^4^Hunter Medical Research Institute, Newcastle, NSW, Australia; ^5^Department of Psychology, National Cheng Kung University, Tainan, Taiwan; ^6^Institute of Allied Health Sciences, National Cheng Kung University, Tainan, Taiwan; ^7^Department and Institute of Public health, National Cheng Kung University, Tainan, Taiwan

**Keywords:** aging, working memory, DWI, arterial pulsatility, cardiovascular risk factors

## Abstract

Aging is associated with an increased prevalence of vascular health conditions that are linked to a disruption in the cerebral vasculature and white matter microstructural organization. In people with cardiovascular risk factors, increased cerebral arterial pulsatility is associated with poorer white matter microstructural organization and cognitive functioning. This study examines the relationship among arterial pulsatility, white matter microstructural organization, and cognitive ability in a healthy adult lifespan sample. One hundred and eighty-nine adults were divided into a younger adult (<50 years, *n* = 97) and older adult (>50 years, *n* = 92). The latter were further subdivided into two subgroups with (CV+, *n* = 25) and without (CV−, *n* = 67) cardiovascular risk factors. Arterial pulsatility was measured using cardiac-gated phase-contrast flow quantification sequence and three indexes of whole-brain white matter microstructural organization [i.e., fractional anisotropy (FA), radial diffusivity (RaD), mean diffusivity (MD)] were derived from diffusion-weighted imaging (DWI). Cognitive ability was assessed using global cognitive functioning (MoCA) and a measure of working memory [sensitivity (*d′*) from a 2-back task]. Neither the whole group analysis nor the younger adult group showed an association between measures of arterial pulsatility, global white matter microstructural organization, and cognition. In older adults, higher MD and RaD were associated with increased arterial pulsatility and poorer working memory performance. The indirect pathway from arterial pulsatility to working memory performance *via* both MD and RaD measures was significant in this group. Interestingly, a comparison of CV+ and CV− subgroups showed that this mediating relationship was only evident in older adults with at least one CV risk factor. These findings are consistent with cardiovascular risk factors as underlying arterial, white matter, and cognitive decline in cognitively normal older adults.

## Introduction

Aging is associated with both increased prevalence of vascular health conditions (e.g., hypertension, diabetes, atrial fibrillation, hypercholesterolemia) and gradual atrophy of the brain. While atrophy affects both gray and white matter, white matter changes emerge later but progress at a much faster rate than gray matter changes (Courchesne et al., 2000; Ge et al., 2002). White matter structural deterioration is more pronounced in frontal compared to posterior locations (van Swieten et al., 1991; Bennett et al., 2010; Madden et al., 2012). Changes in white matter microstructural organization are inferred from diffusion-weighted imaging (DWI) sequences that measure the random motion of water molecules in cortical tissue (Horsfield and Jones, 2002). Common measures include fractional anisotropy (FA) as well as mean and radial diffusivity (MD, RaD, respectively), which quantify the overall uniformity of diffusion in a vessel and the direction of diffusion, respectively.

Cardiovascular (CV) health plays an important role in the preservation of white matter structural organization in older adults. Changes in the cardiac vasculature, such as reduced CV reactivity and hardening of arterial walls (arteriosclerosis) are associated with a disruption in white matter macrostructure, including white matter atrophy and accumulation of white matter hyperintensities (Bateman, 2002; Kennedy and Raz, 2009; Mitchell et al., 2011; Fuhrmann et al., 2019). The presence of CV risk factors is also associated with disruption of white matter microstructural organization, as measured by DWI measures, especially in the long fronto-posterior white matter tracts (Li et al., 2015; Habes et al., 2018; Fuhrmann et al., 2019).

Deterioration of white matter macrostructural and microstructural organization is also associated with cognitive decline in older adults (Breteler et al., 1994; Arvanitakis et al., 2016; Yang et al., 2017) and is more severe in the presence of vascular risk factors, such as hypertension, high blood cholesterol and diabetes (Li et al., 2015; Spielberg et al., 2017; Biessels and Despa, 2018; Kong et al., 2020). The decline in white matter microstructural organization mediates the relationship between vascular risk factors and cognitive performance (Oberlin et al., 2016), as well as the relationship between age and cognitive performance (Jolly et al., 2016, 2017), potentially by reducing the efficiency of neural communication across long fiber tracts (Nilsson et al., 2014). These findings suggest that both cognitive decline and white matter deterioration in older adults may arise from a sub-clinical pathological process of cardiovascular origin.

Cerebral blood flow (CBF) is believed to play a key mediating role in the link between CV risk factors and white matter structural organization (Parkes et al., 2004; Aslan et al., 2011). Age-related reduction in CBF is closely linked to changes in neurovascular coupling and metabolism (Chen et al., 2011). As arterial flow is pulsatile, it causes periodic increases in blood volume into the intracranial cavity, which is balanced by cerebrospinal fluid and venous outflow. Vascular risk factors can impact arterial structural integrity by increasing arterial stiffness (i.e., arteriosclerosis) and restricting blood flow (i.e., atherosclerosis), mechanisms that damage the endothelium (Bateman, 2002; Bateman et al., 2008; Mitchell et al., 2011; O’Rourke et al., 2011). These changes in arterial structure can impact CBF balance by increasing the pulsatile properties of the arterial inflow and/or reducing the volume of venous outflow. Arterial pulsatility, a measure of the pulsatile properties of arterial inflow derived from a cardiac-gated magnetic resonance imaging (MRI) scan, is increased in idiopathic dementia (Bateman, 2002) and early vascular dementia (Bateman, 2004), both of which are characterized by the increased presence of white matter hyperintensities. Moreover, consistent with a link between arterial, white matter and cognitive health in older adults, CV fitness is positively associated with better arterial health (Chapman et al., 2013; Crichton et al., 2014; Zimmerman et al., 2014), white matter structure (Johnson et al., 2012; Burzynska et al., 2014; Fletcher et al., 2016) and cognitive ability, and specifically working memory and processing speed (McAuley et al., 2004; Fabiani et al., 2014; Zimmerman et al., 2014; Gardener et al., 2015; Oberlin et al., 2016).

In community-dwelling older adults, whole-brain white matter microstructural organization (measured using RaD) was positively associated with both global cognitive ability [measured using the Montreal Cognitive Assessment (MoCA; Jolly et al., 2016)] and a measure of executive functioning (mixing cost measured using a task-switching paradigm; Jolly et al., 2017). Whole-brain RaD mediated the relationship between age and cognitive ability, but age did not mediate the relationship between RaD and cognitive ability, suggesting that white matter microstructural organization is a better predictor of cognition than age, *per se* (Jolly et al., 2016). Increased arterial pulsatility was significantly correlated with disruption in whole-brain white matter microstructural organization (i.e., reduced FA, increased RaD), and this relationship remained significant when controlling for age (Jolly et al., 2013). Importantly, the relationships between arterial pulsatility, white matter microstructural organization, cognition, and age were statistically significant *only* in a subgroup of participants (60%) who reported one or more CV risk factors (e.g., hypertension, high blood cholesterol, and atrial fibrillation). These findings suggest that the presence of modifiable CV risk factors may at least partially account for the cognitive and brain changes typically attributed to increasing age. Therefore, efficient treatment of CV risk factors may hold the key to delaying or preventing cognitive decline and brain structural changes in otherwise healthy older adults (Hajduk et al., 2013; Elias et al., 2018; Williamson et al., 2019; Peters et al., 2020).

Although Jolly et al. (2013, 2016, 2017) provided evidence for a relationship between age, cerebral arterial pulsatility, white matter structure, and cognitive performance, the cohort was all over 45 years and biased towards people with CV risk factors. Specifically, half the cohort was recruited from the caseload of a clinical neurologist based on the presence of mild to moderate white matter hyperintensities in their clinical MRI.

In the current study, we sought to replicate and extend the above findings in a larger and more representative sample of older adults and compare the relationships between arterial, white matter structure and cognition in young and older adults, using both a test of global cognitive ability (MoCA) and the 2-back task that targets working memory ability. Working memory plays a critical role in many areas of cognition (e.g., language, maths; Baddeley, 2003; Raghubar et al., 2010) and everyday life activities (e.g., planning what to do and when to do it; Cohen and Conway, 2007; Kane et al., 2007) and is highly sensitive to aging and pathological cognitive decline (Saunders and Summers, 2011; Garcia-Alvarez et al., 2019; Yaple et al., 2019).

Specifically, we sought to examine whether the relationship between arterial pulsatility, whole-brain white matter microstructural organization and both global cognitive and working memory ability is evident in both young and old adults. We also examined whether the relationship between arterial, white matter, and cognitive measures was dependent on the presence of one or more CV risk factors (CV+/CV−). We hypothesized that global cognition and working memory ability will vary with age as well as indices of whole-brain white matter microstructural organization (FA, MD, or RD). Based on Jolly’s findings, we expected that these variables would be more strongly or exclusively related to the presence of one or more cardiovascular risk factors. Specifically, we expected that the whole-brain white matter microstructural organization will mediate the relationship between arterial pulsatility index (AP) and working memory capacity in the CV+ subgroup.

## Materials and Methods

### Participants

Two hundred and five participants were recruited *via* advertisements as part of a larger lifespan data set collected at the National Cheng Kung University, Tainan, Taiwan, R.O.C. Medical data, including information on cardiovascular risk factors and mental health status, were collected *via* a self-report questionnaire. Data from 16 participants were removed from analyses: seven because they scored above 13 on the Beck Depression Inventory-II (BDI-II; Beck et al., 1996), and nine due to excessive artifact in their blood flow scans. The remaining participants were divided into two age groups (≥50 and <50 years). This resulted in a final sample of 92 older adults (44 male, range = 50.08–78.01 years), of whom 25 reported the presence of one or more cardiovascular risk factors, and 97 young adults (57 male, range = 20.32–49.95 years). Information about the CV risk factors is reported in Table 3. No participants reported any diagnosis of a psychiatric disorder. Participants completed a test battery including demographic, neuropsychological, and cognitive tasks, as well as imaging protocols. The study complied with the Declaration of Helsinki, and all participants provided written informed consent. The study protocol was approved by the Research Ethics Committee of the National Cheng Kung University, Tainan, Taiwan, R.O.C. Participants were paid $1,500 NTD after completion of the experiment.

**Table 1:** Age group differences on demographic information, cognitive performance, measures of white matter microstructural organization, and arterial pulsatility.

Measure	Younger adults	Older adults	*t*	*p*
Education (years)	15.57 (2.19)	13.96 (2.61)	−4.61**	8.00E-06
Gender (female/all)	40/97	48/92	−1.51	0.13
MET	11.69 (2.35)	8.49 (2.11)	−9.82**	1.29E-18
BDI-II	5.89 (4.52)	4.49 (4.00)	−2.25*	0.03
MoCA	28.06 (1.88)	27.13 (1.82)	−3.46**	6.70E-04
2-back *d′*	2.40 (0.89)	1.52 (0.79)	−7.09**	2.73E-11
FA	0.45 (0.01)	0.43 (0.02)	−7.32**	1.25E-11
MD	7.55E-04 (1.61E-05)	7.66E-04 (2.66E-05)	3.26**	1.39E-03
RaD	5.51E-04 (1.71E-05)	5.69E-04 (2.97E-05)	5.20**	6.88E-07
AP	0.86 (0.23)	0.99 (0.21)	4.17**	4.60E-05

Notes: measures reported as Mean (SD) unless noted. MET, metabolic equivalent score; BDI, Beck Depression Inventory; MoCA, Montreal Cognitive Assessment; FA, fractional anisotropy; MD, mean diffusivity; RaD, radial diffusivity; AP, arterial pulsatility index. *p < 0.05; **p < 0.001.

**Table 2:** Correlations between arterial pulsatility index, white matter microstructural organization, and cognitive performance in each age group.

Age group	Measure	AP	FA	MD	RaD	MoCA
Young	AP					
	FA	-0.130				
	MD	0.066	-0.758**			
	RaD	0.098	-0.901**	0.964**		
	MoCA	-0.154	0.113	-0.033	-0.076	
	2-back *d*′	-0.082	0.086	-0.049	-0.076	0.191
Older	AP					
	FA	-0.176				
	MD	0.249*	-0.901**			
	RaD	0.242*	-0.955**	0.988**		
	MoCA	-0.042	0.050	-0.059	-0.057	
	2-back *d*′	-0.156	0.267*	-0.277*	-0.286**	0.116
Older CV−	AP					
	FA	-0.131				
	MD	0.176	-0.905*			
	RD	0.183	-0.958*	0.988*
	2-back *d*′	-0.203	0.272	-0.253	-0.275
Older CV+	AP
	FA	-0.353
	MD	0.498*	-0.888*
	RD	0.463*	-0.947*	0.988*
	2-back *d*′	-0.087	0.243	-0.357	-0.324

Notes: AP, arterial pulsatility index; FA, fractional anisotropy; MD, mean diffusivity; RaD, radial diffusivity; MoCA, Montreal Cognitive Assessment; CV−, subgroup of older adults without cardiovascular risk factors; CV+, subgroup of older adults with cardiovascular risk factors. *p < 0.05; **p < 0.001. BDI-II, sex, education, and MET were controlled.

**Table 3:** Group differences in demographic information, cognitive performance, measures of white matter microstructural organization, and arterial pulsatility for older adults with and without cardiovascular risk factors.

Measure	CV+	CV−	*t*	*p*	CV Risk Factor	*n*	%
*n*	25	67			
Age (years)	62.09 (6.48)	62.98 (7.15)	−0.54	0.59	Diabetes	7	28
Education (years)	13 (3.25)	14.31 (2.26)	−2.19*	0.03	Hypertension	16	64
Gender (female/all)	9/25	39/67	1.91	0.06	Hyperlipidemia	2	8
MET	8.39 (1.98)	8.53 (2.17)	−0.79	0.78	Hyperglycemia	4	16
BDI-II	5.2 (4.29)	4.22 (3.88)	1.04	0.30	Stroke	1	4
MoCA	26.24 (2.27)	27.31 (1.61)	−1.36	0.18	Stroke	1	4
2-back d’	1.52 (0.71)	1.52 (0.83)	0.02	0.99	Smoking (Past/Present)	8	32
MD	7.67E-04 (2.56E-05)	7.65E-04 (2.71E-05)	0.42	0.68	Untreated risk factors	3	12
RaD	5.71E-04 (2.80E-05)	5.69E-04 (3.05E-05)	0.28	0.78	Untreated risk factors	3	12
AP	1.05 (0.20)	0.97 (0.21)	1.61	0.11	More than 1 CV risk factor	12	48

Notes: measures reported as Mean (SD) unless noted. MET, metabolic equivalent score; BDI, Beck Depression Inventory; MoCA, Montreal Cognitive Assessment; MD, mean diffusivity; RaD, radial diffusivity; AP, arterial pulsatility index; CV−, subgroup of older adults without cardiovascular risk factors; CV+, subgroup of older adults with cardiovascular risk factors. Also included is the number and proportion of CV risk factors amongst the CV+ group. *p < 0.05.

### MRI Protocols

MRI images were acquired on a GE MR750 3 T scanner (GE Healthcare, Waukesha, WI, USA) in the Mind Research Imaging Center at the National Cheng Kung University. High-resolution structural images were acquired using fast-SPGR, consisting 166 axial slices [TR/TE/flip angle, 7.6 ms/3.3 ms/12°; the field of view (FOV), 22.4 × 22.4 cm^2^; matrices, 224 × 224; slice thickness, 1 mm], and the entire process lasted for 218 s.

### Diffusion-Weighted Imaging (DWI)

Diffusion-weighted imaging (DWI) was obtained with a spin-echo-echo planar sequence (TR/TE = 5,500 ms/62–64 ms, 50 directions with b = 1,000 s/mm^2^, 100 × 100 matrices, slice thickness = 2.5 mm, voxel size = 2.5 × 2.5 × 2.5 mm, number of slices = 50, FOV = 25 cm, NEX = 3). Reverse DTI was also acquired for top-up correction in the DTI pre-processing. The acquisition parameters for the reverse DTI were identical to the DTI—the only difference was that there were six directions.

#### DWI Preprocessing

DWI data pre-processing and analyses were carried out using FMRIB’s Software Library (Smith et al., 2004; Jenkinson et al., 2012), based on the processes outlined by Boekel et al. (2015). For each participant and session, DWI data were concatenated and corrected for eddy currents (including top-up correction). Affine registration was used to register each volume to a reference volume (Jenkinson and Smith, 2001). Following registration, a mask was created from a single image without diffusion weighting (b0; *b*-value = 0 s/mm^2^) using the FMRIB’s Brain Extraction Tool (BET; Smith, 2002). Finally, DTIFIT 37 was applied to fit a tensor model at each voxel to derive FA, MD, and RaD measures (Smith et al., 2004).

#### Tract-Based Spatial Statistics

Following pre-processing, tract-based spatial statistics were generated in FSL for the FA, MD, and RaD images (TBSS; Smith et al., 2006). First, the end slices were removed from the FA images by zeroing to reduce the impact of outliers from the algorithm. Second, all FA images were aligned to a 1 mm standard space using non-linear registration to the FMRIB58_FA standard-space image. Following this, affine registrations were used to align images into 1 × 1× 1 mm MNI 152-space, before being skeletonized. To accurately represent the white-matter tracts, the mean skeletonized image was thresholded at FA of 0.2 based on the default recommendations. Lastly, each participant’s FA data were projected onto the mean skeletonized FA image and concatenated. This process was then repeated for MD and RaD images using the tbss_non_FA function.

#### Quantifying Measures of White Matter

Mean FA, MD, and RaD measures were calculated from whole-brain skeletonized images. FA denotes the degree of anisotropic diffusion of water molecules within the tissue, MD measures the degree of directional diffusivity, and RaD measures the diffusion orthogonal to the principal direction of the vessel (Basser and Jones, 2002; Song et al., 2003). High FA scores as well as low RaD scores are associated with restricted linear diffusion and are interpreted as representing more organized, and by extension, stronger white matter tracts.

### Arterial Blood Flow

Measurements of arterial blood flow were obtained using a retrospectively cardiac-gated phase-contrast flow quantification sequence (TR = 8.5 ms; TE = 4.9 ms; slice thickness = 5 mm; matrix = 256 × 256). To measure the arterial flow, a section plane was positioned to intersect the basilar artery and the cavernous portion of the internal carotid arteries at the base of the skull, as defined by Bateman (2002). Arterial blood flow was calculated using region of interest (ROI) analyses in Medviso Segment version 2.0 R5165 (Heiberg et al., 2010). An ROI was defined around the basilar and carotid arteries to measure the total arterial inflow. Before measuring blood flow, the ROI was baseline corrected against a reference ROI of 1.5 cm^2^ on the brainstem. Arterial pulsatility was derived as the difference between the maximum and minimum flow divided by the mean flow across the ROI (see Jolly et al., 2013, 2016).

### Cardiorespiratory Fitness

To estimate cardiorespiratory fitness, we used a metabolic equivalent score (MET; Jurca et al., 2005) as an estimate of VO_2_max. This score was derived from anthropometric variables including sex, age, body mass index (BMI), resting heart rate (RHR), and self-reported habitual physical activity (Act) levels. The MET was calculated by an equation as follows:

(1) MET=2.77*Gender−0.1*Age−0.17*BMI−0.03*RHR+1*Act+18.07

where Gender: 1 = male and 0 = female and Act = 0, 0.32, 1.06, 1.76, and 3.03 for five different weekly exercise intensity levels. Higher numbers represented higher exercise intensity.

### Cognitive Tasks

In this article, we report cognitive results from the Montreal Cognitive Assessment (MoCA; Nasreddine et al., 2005), a measure of global cognitive functioning, and the 2-back task, a measure of working memory.

**Figure 1:**
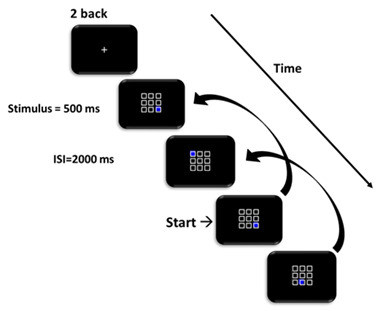
Schematic of the 2-back task. Participants responded whether the position of the blue square matched the position on two trials prior.

We used the version of the 2-back working memory test developed by Jaeggi et al. (2008). On each trial, a 3 × 3 square grid was presented and a randomly selected box was blue (Figure 1). Participants were asked to memorize the last two locations of the blue box. If the location of the blue box on the current trial (*n*) was the same as that two trials back (*n*-2), participants were asked to press the “F” button with their left index finger; if it was in a different location, participants were to press the “J” button with their right index finger. The blue stimulus was displayed for 500 ms and participants were required to respond within a 2,000 ms inter-stimulus interval (ISI). Participants completed four blocks of 21 trials: the first block was task practice and feedback was provided, the remaining three blocks formed the data used in the analyses. The experiment lasted approximately 20–30 min.

The main outcome measure was sensitivity (*d′*) and was calculated based on the hit rate (H) and false-alarm (F) rate using the formula:

(2) $$d^{\prime }=Z(H)-Z(F)$$

where *Z* refers to the *z* score of the normal distribution.

### Data Analyses

To compare with the findings by Jolly et al. (2013, 2016), we used similar analyses to those reported in the original article. However, we transformed all raw data into Z scores before the statistical tests of correlations and mediations. Descriptive, correlation, and regression analyses were run in IBM SPSS v22 (IBM, Armonk, NY, USA).

Pearson correlations were used to examine the relationships between age, arterial pulsatility, and measures of white matter microstructural organization (FA, MD, RaD) on cognitive ability (MoCA score, 2-back *d′*). Familywise error rate correction was used as specified in relevant table legends. For the correlations, we controlled for BDI-II, sex, education, and MET score.

Following this, we examined the associations between arterial pulsatility and white matter measures in younger and older age groups separately. To examine the impact of CV risk factors, we further divided the older group into subgroups based on the self-reported CV risk factors (with risk factors = CV+; without = CV−).

We used mediation analyses to examine whether the relationship between arterial pulsatility and each cognitive ability score was mediated by white matter microstructural organization (i.e., FA, MD, RaD) first in the entire older adult group and then in CV+ and CV− older subgroups. BDI-II, sex, education, and MET score were also controlled in all mediation models. For the mediation analysis, we used the PROCESS macro (Hayes, 2017), which estimates path effects with ordinary least-squares regression. We used the methodology specified by Hayes (2009) to examine the relationships between total, direct and indirect effects, in which an indirect relationship can be established between a predictor and outcome, even in the absence relationship between these two variables. The significance of direct or indirect effects was assessed with a 95% confidence interval. To estimate confidence intervals, we used a bias-corrected method with the percentile bootstrap estimation approach, which implemented 5,000 bootstrap iterations.

## Results

### Age Group Differences

As shown in Table 1, the younger group had, on average, two more years of education than the older group, but also reported more depressive symptoms on the BDI-II. The MET score, an estimate of VO_2_max, was also higher in the younger group. On the cognitive tasks, the younger group scored higher on the MoCA and the 2-back task than the older group. The younger group also had higher FA, lower MD, and RaD, and lower arterial pulsatility scores than the older group.

### Relationship Between Arterial Pulsatility, White Matter Microstructural Organization and Cognitive Ability Within Age Groups

When examined across the entire sample, there were no significant relationships between measures of arterial pulsatility, white matter organization, and cognitive ability (all *p* > 0.05). Following this, we examined these relationships within each age group (Table 2).

Within each age group, measures of white matter microstructural organization were highly intercorrelated. In the younger group, these measures did not significantly correlate with arterial or cognitive variables, nor did the latter correlate with each other. In contrast, in the older group, all DWI measures significantly correlated with scores on the 2-back task (FA: *r* = 0.27; MD: *r* = −0.28; RaD: *r* = 0.29), but not the MoCA. Also, MD and RaD were positively associated with the arterial pulsatility index (both *r* > 0.24).

### The Indirect Effect of Arterial Pulsatility on the Link Between White Matter Microstructural Organization and Cognitive Ability

**Figure 2:**
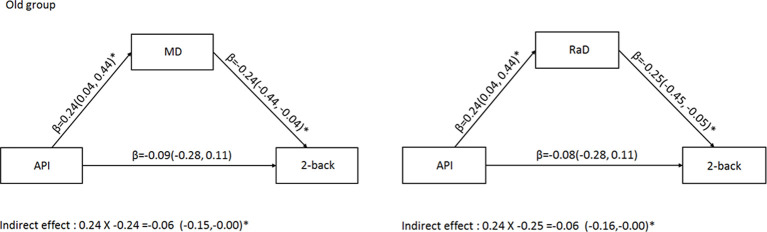
Mediation analysis for the older adult group examining the indirect relationship between arterial pulsatility and 2-back *d*′ when MD (left) or RaD (right) were included as a mediator. Comparisons in the models controlled for BDI-II sex, education, and MET. *Significant 95% confidence intervals.

To assess whether arterial pulsatility had an indirect effect on working memory performance *via* its impact on whole-brain white matter microstructural organization, we ran two mediation models with arterial pulsatility index as the predictor of 2-back performance, and either MD or RaD as the mediator, in the older age group. Figure 2 shows that there was a small, but significant indirect effect between arterial pulsatility and 2-back *d′*, for both MD and RaD mediators (*β* = −0.06).

### Effect of Cardiovascular Risk Factors Present in the Older Adult Group

To identify whether the indirect effect of arterial pulsatility on working memory performance was driven by the presence of CV risk factors in some older adults, we reran the mediation analyses in CV+ and CV− subgroups. As shown in Table 3, only 27% of older adults reported one or more cardiovascular risk factors. CV+ and CV− groups were highly comparable, with the only significant difference being that the CV− group had, on average, one extra year of education.

**Figure 3:**
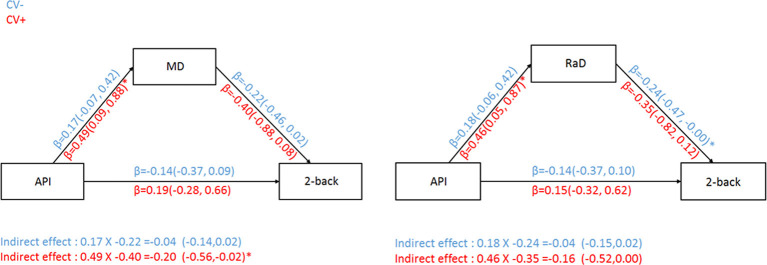
Relationship between arterial pulsatility and 2-back *d*′ with MD (left) or RaD (right) as a mediator for both CV risk groups. CV− = subgroup of older adults without cardiovascular risk factors; CV+ = subgroup of older adults with cardiovascular risk factors. Comparisons in the models controlled for BDI-II, sex, education, and MET. *Significant 95% confidence intervals.

In both MD and RaD models, arterial pulsatility was significantly associated with white measure microstructural organization measures for the CV+ group only (Figure 3). However, the indirect pathway from arterial pulsatility to working memory ability *via* white matter microstructural organization was only significant for MD and only in the CV+ group (Figure 3).

## Discussion

This study examined whether white matter microstructural organization and arterial pulsatility can predict cognitive performance in community-based adults with and without the presence of CV risk factors. When examining these relationships across the adult lifespan, there were no significant relationships between arterial pulsatility, white matter, and cognition. However, consistent with Jolly et al. (2013, 2016), these relationships were present in the older age group (50–78 years), and more strongly in the subgroup of older adults that had one or more CV risk factors. In older adults, increased arterial pulsatility was associated with poorer white matter microstructural organization, which was in turn correlated with worse working memory performance, consistent with Oberlin et al. (2016). The mediation models showed that, for the older adults, there was a small indirect effect of arterial pulsatility on 2-back accuracy with either MD or RaD as the mediator. This effect was specific to the CV+ group, especially when using MD as a mediator. In contrast, there was no indirect effect found in the CV− group for either model.

The association between CV risk factors and white matter structural changes with aging is well established (van Swieten et al., 1991; Johnson et al., 2012). The arterial pulsatility index refers to the balance between arterial inflow and venous and CSF outflow in the cerebral aqueduct. This declines with increasing age and more so in the presence of CV risk factors. For instance, high arterial pressure indicates a greater force of inflow, which may outweigh the ability of the outflow to balance the pressure. This force imbalance affects both gray and white matter tissue and has been linked to white matter atrophy and reduced cognitive performance (Mitchell et al., 2011; Fabiani et al., 2014; Katulska et al., 2014). Moreover, changes in arterial pulsatility have been associated with white matter disease (Bateman, 2002), stroke (Webb et al., 2012), and vascular dementia (Bateman et al., 2008).

The present findings replicate the finding by Jolly et al. (2016) that arterial pulsatility is indirectly related to cognitive performance, but only in the presence of CV risk factors. Also consistent with Jolly’s finding, in the absence of CV risk factors, there was no relationship between white matter microstructural organization and cognitive decline in older adults. These findings are consistent with the notion that age-related cognitive decline may be accounted for by the increased prevalence of CV risk factors. Taken together, these findings support the notion that the interaction between arterial pulsatility and white matter microstructural organization may be the pathway that links CV risk factors to cognitive decline. The present findings are also consistent with Oberlin et al. (2016) who showed that white matter microstructural organization mediated the relationship between VO_2max_ and spatial working memory performance of healthy older adults.

The finding that arterial pulsatility impacts cognitive performance *via* white matter deterioration implies a cascade of changes in the cerebrovascular system that are exacerbated by CV risk factors. As discussed earlier, increased arterial pulsatility may disrupt the balance of inflow and outflow in the intracranial cavity effect leading to a pressure imbalance (or pulse wave encephalopathy, Bateman, 2002, 2004). This pressure imbalance may result in white matter macrostructural damage, especially in periventricular areas, but also have downstream effects on the smaller blood vessels throughout the brain, impacting white matter microstructure. The pulsatile energy which is transmitted into the microvasculature is likely to have a disruptive effect on microcirculation, reducing the reactivity of the microvasculature (Mitchell et al., 2005), and accelerating the development of white matter hyperintensities (Mitchell et al., 2011). Tan et al. (2019) using diffuse optical tomography showed that increasing arterial stiffness of cortical arteries was associated with increased white matter lesion load as well as decreased executive function performance.

It is important to note that, in this community sample, only a relatively small percentage (27%) of older adults had one or more CV risk factors. Yet, despite the relatively small group size (*n* = 25), the relationships between AP and MD/RaD were most robust in this CV+ compared to the CV− (*n* = 67) subgroup. The greater percentage of people with CV risk factors in Jolly’s cohort (60%) is likely due to the selection of people with low to moderate white matter hyperintensities. As such, the present sample is more representative of the prevalence of CV risk factors within older adults, especially in an Asian community.

Like most aging studies, the findings are based on a cross-sectional design. It is, therefore, not possible to make strong directional links between the level of arterial pulsatility, white matter microstructural health and cognitive performance, or predict whether the presence of CV risk factors in the older group leads to accelerated cognitive decline. As the data presented here are the first wave of a longitudinal study, our future work will be able to address these questions.

In conclusion, these findings are consistent with a strong link between cardiovascular health and both neural and cognitive changes typically seen with increasing age. These findings reinforce the importance of considering the presence of cardiovascular risk factors when examining the effect of aging on brain structural and cognitive changes in healthy older adults.

## Data Availability Statement

The raw data supporting the conclusions of this article are available upon request for researchers who meet the criteria for access to confidential data.

## Ethics Statement

The studies involving human participants were reviewed and approved by Research Ethics Committee of the National Cheng Kung University, Tainan, Taiwan. The patients/participants provided their written informed consent to participate in this study.

## Author Contributions

AC contributed to the design of the experiment, processing of blood flow sequences, and the design and preparation of the manuscript. FK contributed to the design of the experiment and analyses and preparation of the manuscript. TJ contributed to the processing of the blood flow sequences and preparation of the manuscript. M-HY contributed to the processing of blood flow sequences, data collection and analysis, and preparation of the manuscript. SH contributed to the design of the experiment and analyses, data collection and processing, and preparation of the manuscript.

## Conflict of Interest

The authors declare that the research was conducted in the absence of any commercial or financial relationships that could be construed as a potential conflict of interest.
